# Perceptions of plain packaging and health warnings among university students in Turkey: a survey-based experiment

**DOI:** 10.1186/s12889-023-15637-4

**Published:** 2023-04-28

**Authors:** Asena Caner, Belgi Turan, Mehmet Y. Gürdal, Sibel Güven

**Affiliations:** 1 Department of Economics, TOBB University of Economics and Technology, Ankara, 06560 Turkey; 2 Department of Economics, Bogazici University, Istanbul, 34342 Turkey; 3 The Economic Policy Research Foundation of Turkey (TEPAV), Ankara, 06560 Turkey

**Keywords:** Plain packaging, Youth Smoking, Graphic health warnings, Quitting intentions, Negative affect, Avoidant responses, Turkey, I12, I18, D91

## Abstract

**Background:**

Cigarette pack design plays a crucial role in attracting customers, especially when other marketing methods are limited by policy. University students who engage in casual smoking take the risk of developing an addiction. The objective of this study was to assess the effects of plain packaging (PP) and graphic health warnings (GHWs) on cigarette packages on three outcome variables (negative affect, avoidant responses, and intentions to quit) among ever-smoker university students in Ankara, Turkey, where youth smoking prevalence is high.

**Methods:**

An online survey-based experiment was used to collect data. The respondents were randomly assigned to one of the five conditions that contained images of cigarette packs with specific design elements. Regression analyses (*n* = 623) were used to compare across conditions and to estimate the effects of combined warnings (versus text-only warnings), stronger GHWs (versus old GHWs), and PP (versus branded packages) on the outcome variables, accounting for potential confounders.

**Results:**

Stronger GHWs generated more negative affect (0.31 points out of 5, *p* = 0.010) and avoidant responses (0.42 points out of 5, *p* = 0.002) than old warnings (when brand logos were visible). Plain packages generated more negative affect (0.48 points out of 5, *p* < 0.001) and avoidant responses (0.46 points out of 5, *p* = 0.001) than branded packages (with old warnings). Disentangling the effects of PP and new GHWs revealed that neither had individual differential effect on intentions to quit within 6 months.

**Conclusions:**

Although no differential effect of PP or harsher GHWs was found on intentions to quit when respondents were exposed to images on screen, both design elements were found to be effective in generating negative affect and avoidant responses. More work is needed to design effective tobacco control measures among youth during critical years of tertiary education.

**Supplementary Information:**

The online version contains supplementary material available at 10.1186/s12889-023-15637-4.

## Background

Smoking is a leading preventable cause of death worldwide [1]. Under the Framework Convention on Tobacco Control (FCTC) of the World Health Organization, countries use various measures to curb smoking [1, 2]. Two prominent regulatory measures on the demand side of the market are the requirement of displaying health warnings on cigarette packages and plain packaging (PP).

Tobacco product packaging is an integral part of marketing in building brand awareness and customer loyalty. Therefore, the tobacco industry has historically put substantial effort into branding and package design [3–5]. In contrast, PP aims to prevent tobacco products from signalling misleading information about their characteristics and health effects, and from targeting specific customers. Plain packages have no branding, colours or images; they include only the brand name in a mandated size, and health warnings in text and graphics. PP was first implemented in Australia in 2012, followed by France, the UK, New Zealand, Norway, and Ireland. As of January 2021, it has been enforced in 17 countries including Turkey [1].

PP is expected to alter smoking behaviour through three channels. First, by reducing the appeal of packages [6], PPs might diminish the demand for tobacco products. Images, colours and fonts are important design elements in determining product appeal and perceptions of product quality, and in triggering associations with personality types, social status, femininity and masculinity [6, 7]. Second, by eliminating branding, colours and images, PPs might draw more attention to graphic and text health warnings, thereby reinforcing the potential effect of warnings on altering smoking behaviour [7]. Related evidence suggests that the effect of graphic health warnings (GHWs) depends on their design and that PP increases the salience and effectiveness of GHWs [8]. Moreover, the reduction in appeal and increased health warning effectiveness is generally sustained for up to 12 months after the implementation of PP [9]. Third, by removing misleading labels, such as “light”, “mild”, or “low tar”, PPs reduce the ability of cigarette brands to mislead consumers by downplaying the harmful effects of their products [6].

There is mixed evidence on whether the reduction in appeal, increase in attention to health warnings, and change in perceptions of the health effects (induced by PP) translate into actual changes in smoking behaviour. Some studies have shown that PP is effective in increasing the intention to quit [10, 11]. Other studies, however, did not find any evidence of deterrence, reduction, or cessation of smoking in response to PP [12, 13]. Although systematic reviews tend to support that PP has a deterrent effect on smoking behaviour, this claim warrants further research [14–16].

Earlier research has shown that compared to text-only warnings, graphic health warnings (GHWs) are more effective in attracting attention and increasing the intention to quit [17, 18]. Moreover, stronger and more striking [17, 19, 20], and coloured (compared to black and white) warnings [21, 22] were perceived as more effective. Longitudinal analysis among adolescents revealed that cognitive processing of GHWs is elevated with their introduction; however, the effect diminishes after 5 years, suggesting that messages need to be refreshed more frequently [23].

This study is also related to the strand of literature that evaluates the effects of different combinations of GHWs and PPs in altering smoking-related perceptions and behaviours. GHWs have been found to be more effective than PP in impacting cigarette cravings, evoking fear, and increasing thoughts of quitting [13]. Some studies report that the combination of PPs and GHWs is more effective than either intervention alone [24, 25], while others report no significant combined effect of PPs and GHWs [13].

In Turkey, Law No. 4207 passed in 1996 required a text warning about the detrimental health effects of smoking (“Disclaimer: Harmful to Health”) to be placed on cigarette packs [26]. Over the years, text warnings were diversified and required to cover a larger part of the pack. The regulation in 2005 required, in addition to keeping the earlier disclaimer, the general warnings “Smoking/Tobacco Kills” and “Smoking/Tobacco causes serious harm to you and those around you” and a text warning to be displayed on cigarette packs [27].

Until 2010, cigarette packs sold in Turkey had only text warnings. Brand logos were visible and packs were printed in brand-specific colours. With the regulation in 2010, combined health warnings were introduced by adding graphic health warnings (GHWs) to text warnings that were already on cigarette packs [28]. The GHWs printed on the packages were selected from pictures in the archive developed by the European Union.

Law No. 7151 in November 2018 and the subsequent regulations in March 2019 [29, 30] introduced PP and stronger warnings, to be effective by the beginning of 2020. All cigarette packs were required to have a black background; the logo, symbol, or any other kind of branding could not be placed on the packages. Brand names were required to be printed in a standard format on all packs. The combined health warnings on packs were also replaced by stronger text warnings with accompanying and more striking GHWs.

In Turkey, only a few studies have been conducted on GHWs or PP. Related studies in the literature have focused on the thoughts and perceptions of high school or university students about the effectiveness of GHWs and PP in deterring consumers or encouraging them to quit smoking [31–35]. To the best of our knowledge, the effectiveness of PP and GHWs in Turkey has not been studied using an experimental approach such as the one used in this study.

In this study, we aimed to estimate the impact of PP and stronger GHWs on three outcome variables (negative affect, avoidant responses, and intentions to quit) in Turkey using a survey-based experiment that included 623 ever-smokers from four universities in Ankara. We specifically focused on university students for several reasons. First, evidence suggests that health warnings and graphics have differential effects depending on the age profile [36]; therefore, pooling individuals with heterogeneous responses, as done in cross-sectional studies [9, 37, 38], might dilute the true policy effects. Second, although there are many studies on the impact of package characteristics on adult smokers, evidence specifically pertaining to young adults and adolescents is relatively sparse, especially in developing countries [6, 13, 39]. Third, as young adult smokers have a shorter history of smoking, they might still be experimenting, and they might see little risk in smoking a few more cigarettes [40]; therefore, the marginal effect of these policies might be more pronounced in this age profile. Moreover, university students represent a vulnerable group because many smokers begin smoking regularly during tertiary education owing to peer effects and social pressure.

This paper contributes to the literature, first, by providing evidence from an upper-middle income country with a high smoking prevalence rate (at around 28% of the population over age 15, constituting the second highest rate among OECD countries [41]). As of 2017, tobacco use was a leading risk factor associated with the highest number of deaths and disabilities in Turkey [42]. Secondly, a survey-based experimental design was used, in contrary to extant work in Turkey. The experiment focused on university students, a vulnerable group, almost all of whom were aware that smoking cigarettes is harmful to health.

## Methods

### Study design, setting, and participants

We studied whether PP and new harsher health warnings increased negative affect, avoidant responses, and quitting intentions. We used a survey experiment in which we randomly assigned respondents to one of the five experimental conditions (used as control or treatment groups). The experimental conditions were designed by the researchers by combining the old or currently used package design elements, with the intention of disentangling the individual differential effects of the design elements on negative affect, avoidant responses, and quitting intentions.

An online survey experiment was conducted among university students in Ankara, the capital city of Turkey, in June-July 2021. In the 2020–2021 academic year, there were 21 universities and 229,251 university students (110,413 male and 118,838 female) in Ankara, the target population [43].

### Survey instrument

A questionnaire (prepared in SurveyMonkey) was used to collect the data. The questionnaire was developed by the researchers by adopting the questions in internationally validated questionnaires (specifically, the Global Adult Tobacco Survey of the World Health Organization and CDC) [44]. To further validate the questionnaire, a pilot test of the online survey was performed to ensure that the questions were clear and that the survey ran smoothly. The questionnaire was designed so that participants could not be identified by the researchers and remained anonymous regarding the reporting of the results.

The first page of the survey asked whether the respondent had smoked cigarettes. Ever-smokers (defined as current regular, occasional, or past smokers) were directed to the questionnaire that collected data for this study. Permission for the study was obtained from the Human Research Evaluation Board (Ethics Committee) of one of the participating universities prior to the data collection. Informed consent was obtained from all participating students, none of which were under the age of 18.

### Sampling

The administrations of universities with large enrollments were contacted by e-mail or by phone and invited to share the online survey link with their students via their university e-mail addresses. The approval letter from the Human Research Evaluation Board was shared with the participants.

Four universities agreed to participate and share the link to the survey, the informed consent form, and the approval letter of the Ethics Committee with their students. Two reminder e-mails were sent approximately one week and two weeks later. Participation was voluntary and no incentives were provided. The participants completed the questionnaire at their own pace and were permitted to leave the survey at any time without any penalty.

There were 80,568 students in the four universities; however, we did not have access to response rate metrics because we did not know how many students actually received the invitation. A total of 1342 students answered the “ever-smoked cigarettes” question at the beginning of the survey, 853 of whom declared that they were ever-smokers and were directed to the ever-smoker survey. Our analyses used the sample of 623 respondents who answered all questions that were of interest to this study.

### Measurements

The questionnaire collected data on demographics (age, gender, and income), pattern of smoking (age at initiation, whether ever tried to quit, nicotine dependence, and past 30-day consumption), the respondent’s perception that consumption is not high enough to be harmful, and the perception of harmfulness and addictiveness of cigarettes.

For the next set of questions, we used a feature of SurveyMonkey to randomly assign participants to one of the five experimental conditions described below:

- Condition 1: Brand logos + Old text warnings (no pictorial warnings)
- Condition 2: Brand logos + Old text warnings with pictorial warnings
- Condition 3: Brand logos + New text and pictorial warnings
- Condition 4: No brand logos (PP, black background) + Old text and pictorial warnings
- Condition 5: No brand logos (PP, black background) + New text and pictorial warnings

**Fig. 1:**
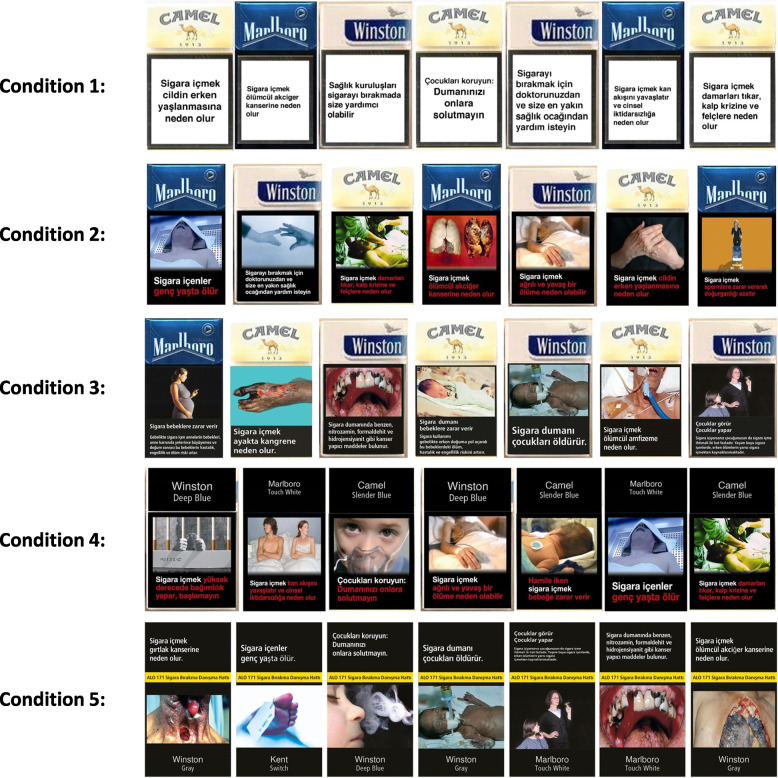
A sample of 7 (out of a total of 14) images of cigarette packs in the randomly assigned conditions

In each condition, images of 14 cigarette packs, specially designed by our research team, were shown to the respondents. Students were asked to examine the images carefully. Each pack had one of the 14 text warnings (old or new) on it. The three most popular brands among youth (Winston, Marlboro, and Camel) were randomly distributed to these 14 packs [34]. Figure 1 shows a sample of images of seven cigarette packs from each condition.

To ensure that the participants were sufficiently exposed to the graphics and warnings, the images were placed on each page with the questions. There was no time limit for answering the questions; therefore, respondents could examine the images as they wished while answering the questions.

### Outcome variables

The outcome variables were *Negative affect, Avoidant responses*, and *Intentions to quit*, (see Additional file 1: Table S.1) adapted from validated scales. The following questions. were asked.

*Negative affect*: “Imagine yourself as a consumer of cigarettes in one of these packs. After looking at the images of cigarette packs, did you feel...” [afraid, angry, annoyed, sad, disturbed, grossed-out, scared, and guilty]. Response choices ranged from 1 to 5, where 1 = Not at all, 5 = Extremely [36]. Each of these eight feelings of negative affect was rated by the respondents from 1 to 5. The *Negative affect* score of the respondent was the average of eight scores (mean = 2.18, SD = 1.08, Cronbach’s α = 0.9189).

*Avoidant responses*: “Imagine that you purchase/smoke cigarettes in one of these packs: How strongly do you agree with the following statements? 1 = Strongly disagree, 5 = Strongly agree: If I were smoking when I see one of these packs, I would stop smoking; I try to cover the pack to avoid seeing it; I try to hide it somewhere I cannot see; I would use a pack cover or a cigarette container in order not to see the pack; I prefer to buy a pack with another look.” [45]. Each of the five statements representing avoidant responses was rated by the respondents from 1 to 5. The *Avoidant responses* score of the respondent was the average of the five scores (mean = 2.23, SD = 1.18, Cronbach’s α = 0.8688).

*Intentions to quit*: “Imagine yourself as a consumer of cigarettes in one of these packs. Please select one of the following alternatives: I am not planning/have no intention to quit; I have an intention to quit, but I do not have a plan; I will quit within a week; I will quit within a month; I will quit within six months; I will quit within a year; I will quit in more than a year [36]. *Intentions to quit* is a dummy variable that takes the value of 1 if the respondent intends to quit within six months or sooner, and 0 otherwise (mean = 9.63%, SD = 29.52).

### Potential confounders

The following variables were used in the analyses: *Age*; *gender* (female or male); *income* (“In an average week, how much money can you freely spend on leisurely activities? Exclude living expenses such as rent, food, transportation etc.: 0 TL, 20–50 TL (2.4–5.9 USD), 50.1–100 TL (5.9–11.8 USD), 100.1–250 TL (11.8–29.4 USD), 250.1–500 TL (29.4–58.8 USD), 500.1–750 TL (58.8–88.2 USD), 750.1–1000 TL (88.2–117.6 USD), and more than 1000 TL (117.6 USD).” *Income* was set equal to the midpoint of the brackets, except for the highest one, which was set equal to 1500 TL (176.5 USD).

*Smoking is addictive* (1 = Definitely disagree, 2, 3, 4, or 5 = Definitely agree); *Cigarette harm perception* (Are cigarettes harmful to health? 1 = Not harmful at all, 2 = Probably not harmful, 3 = Probably harmful, 4 = Absolutely harmful. Those who chose “No opinion” (3 students) were excluded from the sample.)

The other variables used in the analysis were *Age at initiation* (“How old were you when you first tried a cigarette?”); *Quit attempt* (“Tried to quit in the past? 1 = Yes, 0 = No past quit attempts”); *Nicotine dependence* (To be brief, we used only the first question in the Fagerström test for nicotine dependence, which asks how soon after waking up the respondent smokes the first cigarette: 3 = within 5 min, 2 = in 6–30 min, 1 = in 31–60 min, and 0 = after 60 min. This question has been stated in the literature as being sufficient to show the level of nicotine dependence [46]. It was also found to be useful for assessing dependence in the Turkish context [47].)

*Past 30-day consumption* (Average daily cigarette consumption in the past 30 days: None, Fewer than 1, 1, 2–5, 6–10, 11–20, 21–30, or More than 30); *Smoking but not harming self* (“I smoke/used to smoke, but not as much or as often as to harm myself”: 1 = Definitely disagree, 2, 3, 4, or 5 = Definitely agree).

### Statistical analyses

Descriptive statistics were reported for the independent variables. For the outcome variables, the means (or percentage) and 95% confidence intervals were reported across the five conditions to detect any statistically significant differences among conditions in the answers of the respondents. To confirm that randomization across the five conditions was properly performed, *p*-values from F-tests and chi-square tests of equality were used.

Ordinary least squares regressions were used to estimate *Negative affect* and *Avoidant responses*. The linear probability model was estimated for *Intentions to quit*. All of the potential confounders described above were used in these estimations. Analyses were conducted using Stata MP 15.1.

Comparisons across experimental conditions were made by assigning the reference category to the control group (condition 1, 2, 3, or 4) and estimating the relative effect of the treatment group (condition 2, 3, 4, or 5) on the outcome variables. In particular, the effects of:

- combined health warnings relative to text-only warnings (on packages with brand logos) (condition 2 relative to 1),
- new warnings relative to old warnings (on packages with brand logos) (condition 3 relative to 2),
- PP relative to branded packages (when old warnings were displayed) (condition 4 relative to 2),
- PP relative to branded packages (when old warnings were displayed) (condition 5 relative to 3),
- new warnings relative to old warnings (on plain packages) (condition 5 relative to 4) were estimated.

## Results

### Descriptive analyses

Table 1 shows that a slight majority of ever-smokers (56%) were male. On average, initiation of smoking occurred during high school (around age 16) and the standard deviation (2.63) implies that around 70% of initiation dates fall in high school or early college years. Participants indicated, on average, that their weekly budget available for leisurely activities was 486.83 TL (57.28 USD) corresponding to around 30–35 packages of cigarettes, based on 2021 prices. Previous attempts to quit were highly common among participants (56% of ever-smokers). Approximately 10% of the participants reported having smoked more than one pack a day in the last month, while 25.52% reported no cigarette consumption during that period. Participants demonstrated almost full agreement with the statements that smoking is addictive (55.86% definitely agreed) and is harmful to health (85.55% said absolutely harmful). About half of ever-smokers thought they were harming themselves while smoking.

**Table 1:** Descriptive statistics (mean and standard deviation, or frequencies)

Panel A					
	**Mean**	**SD**	**Min**	**Max**	***p*-value**
**Age**	21.96	2.41	18	31	0.59
**Income**	486.83	459.25	0	1500	0.99
**Age at initiation**	16.16	2.63	7	26	0.32
**Quit attempt**	0.56	0.50	0	1	0.53
***Negative affect***	2.18	1.08	1	5	< 0.01
***Avoidant responses***	2.23	1.18	1	5	< 0.01
***Intention to quit (%)***	9.63	29.5	0	100	0.15
**Panel B**					
**Frequencies (%)**					***p*-value**
**Gender**					0.99
Male	56.02				
Female	43.98				
**Nicotine dependence**					0.13
3: within 5 min	8.67				
2: in 6–30 min	19.10				
1: in 31–60 min	14.77				
0: after 60 min	57.46				
**Past 30-day consumption**					0.17
None	25.52				
10 cigarettes/day or less	40.45				
More than 10/day	34.03				
**Smoking is addictive**					0.44
1: Definitely disagree	2.41				
2: Disagree	5.94				
3: Neither agree, nor disagree	13.32				
4: Agree	22.47				
5: Definitely agree	55.86				
**Cigarette harm perception**					0.40
1: Not harmful at all	0.96				
2: Probably not harmful	0.16				
3: Probably harmful	13.32				
4: Absolutely harmful	85.55				
**Smoking but not harming self**					0.57
1: Definitely disagree	21.35				
2: Disagree	29.21				
3: Neither agree, nor disagree	21.99				
4: Agree	17.50				
5: Definitely agree	9.95				

In Panel A, *p*-value refers to the *p*-value of the F-test where H_0_ is all slope coefficient estimates are zero in the ordinary least squares regression of the variables listed in the table on condition dummy variables. In Panel B, *p*-values of chi-square tests for equality of percentages across conditions are reported. *SD* Standard deviation, *Min* minimum, *Max* maximum

Table 1 also provides evidence that randomization across the five experimental conditions was performed properly. The last column of the table reports the *p*-values of the tests for the equality of means (or percentages) across conditions. For almost all the independent variables, the *p*-values were large, indicating that the null hypothesis of equality could not be rejected. Low *p*-values were observed for two outcome variables.

**Fig. 2:**
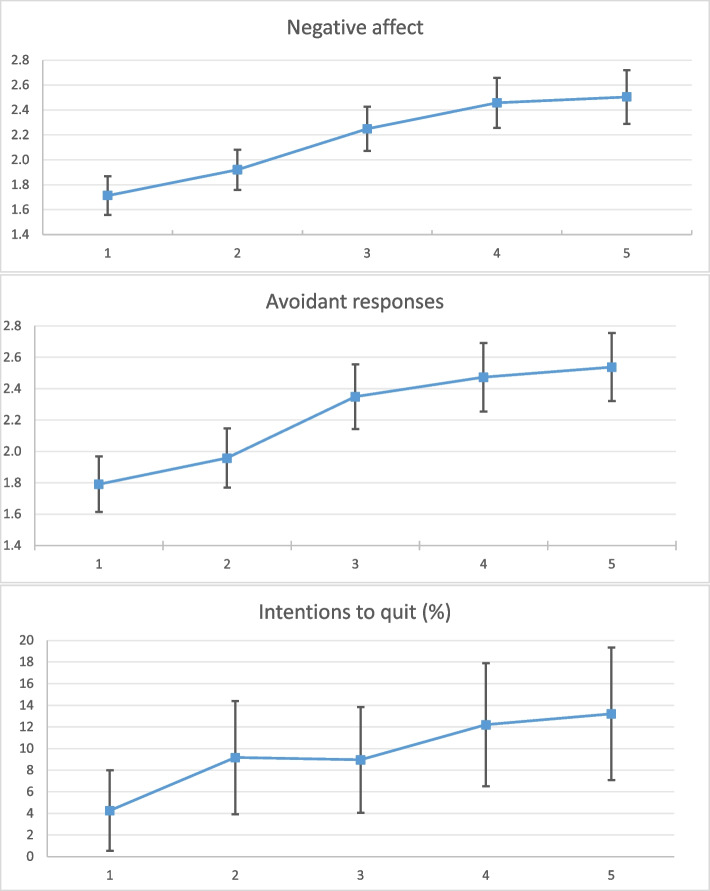
Mean and 95% confidence interval values for *Negative affect, Avoidant responses,* and *Intentions to quit*, by condition

Figure 2 shows the mean values and 95% confidence intervals for *Negative affect, Avoidant responses,* and *Intentions to quit*, by condition. Evidently, in the first and second graphs, conditions 1 and 2 were separated from conditions 3, 4, and 5, which generated more negative affect and stronger avoidant responses on average. In contrast, *Intentions to quit* reported in the five conditions could not be ranked, as the 95% confidence intervals overlapped and there was no statistically significant difference across conditions. (For summary statistics on the underlying variables used to construct the outcome variables, please refer to Table S1 in the Additional file 1).

### Regression analyses

Table 2 shows the regression estimates of the effects of different package designs on negative affect, avoidant responses, and intention to quit. Model 1 included only dummy variables (fixed-effects) for conditions and Model 2 added other correlates to Model 1.

**Table 2:** Regression models explaining the three outcome variables

	**(1)**	**(2)**	**(3)**	**(4)**	**(5)**	**(6)**
**Outcome Variable:**	**Negative affect**		**Avoidant responses**		**Intentions to quit**	
	**Model 1**	**Model 2**	**Model 1**	**Model 2**	**Model 1**	**Model 2**
**Condition 1**	0	0	0	0	0	0
**Condition 2**	0.21*	0.21*	0.17	0.17	0.049	0.039
**Condition 3**	0.54***	0.53***	0.56***	0.59***	0.047	0.047
**Condition 4**	0.74***	0.70***	0.68***	0.62***	0.079**	0.065**
**Condition 5**	0.79***	0.75***	0.75***	0.70***	0.089**	0.065*
**Age**		0.033*		-0.015		-0.0072
**Gender:** Male		0		0		0
Female		0.32***		0.50***		-0.027
**Income (in ‘000)**		-0.042		0.032		-0.032
**Age at initiation**		0.041**		0.033**		-0.0012
**Quit attempt**		0.069		0.083		0.019
**Nicotine dependence:** 0: > 60 min		0		0		0
1: in 31–60 min		-0.082		-0.17		-0.044
2: in 6–30 min		-0.38***		-0.18		-0.068**
3: within 5 min		-0.38**		-0.057		-0.080**
**Past 30-day consumption:** None		0		0		0
10/day or less		-0.38***		-0.39***		-0.15***
More than 10/day		-0.32**		-0.56***		-0.11***
**Smoking is addictive**						
Disagree/Definitely disagree		-0.081		-0.19		0.017
Neither agree, nor disagree		0		0		0
Agree/Definitely agree		0.11		-0.013		0.015
**Cigarettes absolutely harmful**		0.24**		0.068		0.057**
**Smoking but not harming self:**						
Disagree/Definitely disagree		-0.032		0.048		0.019
Neither agree, nor disagree		0		0		0
Agree/Definitely agree		-0.098		0.073		0.030
**Constant**	1.71***	0.33	1.79***	1.66***	0.043**	0.29**
**N**	623	623	623	623	623	623

Columns in the table show estimates from different regressions. Coefficient estimates for reference categories are zero. Model 1 included only the dummy variables for conditions. Model 2 added other control variables. Ordinary least squares was used to estimate regressions in columns (1)-(4). Linear probability model was used to estimate regressions in columns (5)-(6). Condition 1: Brand logos + text, Condition 2: Brand logos + old text + old pictures, Condition 3: Brand logos + new text + new pictures, Condition 4: PP + old text + old pictures, Condition 5: PP + new text + new pictures. Negative affect was the average of scores in eight questions (1 to 5). Avoidant responses was the average of scores in five questions (1 to 5). Intentions to quit was a dummy variable that was 1 if the respondent intended to quit within six months or sooner, and 0 otherwise. The categories of ‘Past 30-day consumption’, ‘Smoking is addictive’, and ‘Smoking but not harming self’ were condensed to present the results more concisely. Since a large majority of the responses to harm perception question was ‘absolutely harmful’, a new variable ‘Cigarettes absolutely harmful’ was created as a dummy variable equal to 1 if ‘absolutely harmful’ was chosen, 0 if one of the other three responses was chosen. *** *p* < 0.01, ** *p* < 0.05, * *p* < 0.10

The first important result was that the coefficient estimates of the condition dummies were very similar in the two models. Estimates in Column [2] showed that females, on average, had 0.32 points (out of 5, *p* < 0.001) higher negative affect than males, and those who believed that cigarettes are absolutely harmful had 0.24 points (*p* = 0.018) higher negative affect, irrespective of the package design. Nicotine dependence and consumption intensity were negatively associated with negative affect, meaning that light users of cigarettes experienced more negative feelings regardless of the package design. Those who initiated smoking at older ages (who had a shorter history of smoking) also experienced more negative feelings (*p* = 0.011). However, income and past quit attempt were not found to be significantly correlated with negative affect.

The estimates in Column [4] show that females had 0.50 points (out of 5, *p* < 0.001) higher avoidant responses than males, irrespective of the package design. Respondents with higher past 30-day consumption showed significantly less avoidant behaviour and those who started smoking late (*p* = 0.043) had more avoidant behaviour. Age, income, perception of harm about cigarettes, and past quit attempts did not have a statistically significant relationship with avoidant responses.

Column [6] shows that once the other correlates of smoking behaviour are controlled for, gender, age, income, age at initiation, and past quit attempts were not found to be associated with the intention to quit within six months or sooner. On the other hand, stronger harm perception of cigarettes was positively correlated (*p* = 0.021) with quitting intentions. Higher nicotine dependence and past 30-day consumption intensity were negatively correlated with quitting intentions.

### Comparisons across conditions

Table 3 compares the effects of the package design elements (in Model 2) on the three outcome variables. The effect of combined health warnings relative to text-only warnings (on packages with brand logos) on negative affect was estimated as 0.21 points out of 5 (*p* = 0.066) (condition 2 versus 1, in column (1)). The effect of new warnings relative to old warnings (on packages with brand logos) (condition 3 versus 2, in column (2)) on negative affect was 0.31 points out of 5 (*p* = 0.010), and on avoidant responses was 0.42 points out of 5 (*p* = 0.002).

**Table 3:** Regression models for the three outcome variables, comparing responses across experimental conditions

	**(1)**	**(2)**	**(3)**	**(4)**
Outcome Variable: Negative Affect				
Condition 1	0	-0.21*	-0.53***	-0.70***
Condition 2	0.21*	0	-0.31***	-0.48***
Condition 3	0.53***	0.31***	0	-0.17
Condition 4	0.70***	0.48***	0.17	0
Condition 5	0.75***	0.53***	**0.22**	**0.051**
Outcome Variable: Avoidant Responses				
Condition 1	0	-0.17	-0.59***	-0.62***
Condition 2	**0.17**	0	-0.42***	-0.46***
Condition 3	0.59***	0.42***	0	-0.031
Condition 4	0.62***	0.46***	0.031	0
Condition 5	0.70***	0.53***	**0.11**	**0.078**
Outcome Variable: Intentions to Quit				
Condition 1	0	-0.039	-0.047	-0.065**
Condition 2	**0.039**	0	-0.0083	-0.026
Condition 3	0.047	**0.0083**	0	-0.018
Condition 4	0.065**	**0.026**	0.018	0
Condition 5	0.065*	0.027	**0.018**	**0.00044**
N	623	623	623	623

Columns in the table show estimates from different regressions. Estimates that were used to compare outcome variables across conditions are typed in bold. Coefficient estimates for reference categories are zero. Four versions of Model 2 were estimated, using in each version a different condition as the reference category. Condition 1: Brand logos + text, Condition 2: Brand logos + old text + old pictures, Condition 3: Brand logos + new text + new pictures, Condition 4: PP + old text + old pictures, Condition 5: PP + new text + new pictures. *** *p* < 0.01, ** *p* < 0.05, * *p* < 0.10

In addition, the effect of PP relative to branded packages (when old warnings were displayed) (condition 4 versus 2, in column (2)) on negative affect was 0.48 points out of 5 (*p* < 0.001), and on avoidant responses was 0.46 points out of 5 (*p* = 0.001). Regression analyses did not yield any statistically significant effect of PP relative to branded packages (when old warnings are displayed) (condition 5 versus 3, in column (3)), or new warnings relative to old warnings (on plain packages) (condition 5 versus 4, in column (4)) on the three outcome variables. The results are summarized in Table 4.

**Table 4:** Effects of package design elements on outcome variables estimated through regression analyses by comparing across experimental conditions (Based on the results in Table 3)

Condition 1 vs. Condition 2 (Column (1)):	Combined health warning compared to text-only warnings (on packages with brand logos)	**More** negative affect (at 10% statistical significance)
Condition 2 vs. Condition 3 (Column (2)):	New warnings compared to old warnings (on packages with brand logos)	**More** negative affect and avoidant responses
Condition 2 vs. Condition 4 (Column (2)):	PP compared to branded packages (when old warnings are displayed)	**More** negative affect and avoidant responses
Condition 3 vs. Condition 5 (Column (3)):	PP compared to branded packages (when new warnings are displayed)	No effect on any of the outcome variables
Condition 4 vs. Condition 5 (Column (4)):	New warnings compared to old warnings (on plain packages)	No effect on any of the outcome variables

## Discussion

In this study, unlike any of the related earlier studies in Turkey, a survey-based experiment was used to randomly assign participants to control and treatment groups (eliminating the bias due to self-selection into groups), with the aim of estimating the differential effects of PP and GHWs on three outcome variables. It was found that among university students PP and stronger GHWs were effective in generating negative affect and avoidant responses among ever-smokers of cigarettes, in a setting where almost all of the participants were aware that smoking cigarettes is harmful to health. Earlier studies have reported similar findings for PP [35, 48, 49] and GHWs [14, 36, 50].

At the time of the switch to PP, Turkey replaced the old GHWs on cigarette packs with new and stronger ones; therefore, it is important to disentangle the two effects [38], in order to know whether one supplemented and reinforced the impact of the other. In this study, which had a between-subjects design, no statistically significant effect of PP relative to branded packages (when old warnings are displayed), or new warnings relative to old warnings (on plain packages) was found on any of the three outcome variables. In other words, no individual differential effect of PP or new warnings was detected.

Comparisons of average intention to quit across experimental conditions showed that the disentangled differential effects of PP and stronger GHWs on intention to quit were statistically zero. This implies that further measures and tools are necessary for effective tobacco control. The findings in the related literature are mixed [37, 38]. Our results echo those of studies that reported a statistically insignificant effect on the intentions to quit [12, 13, 33, 36]. However, it should also be noted that the result pertains to the exposure to images in an online experiment, which may not be realistic enough to generate such a hard outcome as quitting intentions.

It is noteworthy that the differential effect of GHWs (relative to text-only warnings) (condition 1 versus 2) was small on negative affect and statistically zero on the other two outcome variables. Such a finding was unexpected in light of earlier studies [17, 39] which showed that compared to text-only warnings GHWs are more effective; however, it can be explained as a “wear out” effect or desensitization to current packaging warnings [51]. Combined (text + graphic) health warnings have been on cigarette packs since 2010 in Turkey; hence, smokers have been exposed to them for quite a while, especially if they initiated smoking at younger ages. Our regression estimates showed that negative affect and avoidant responses were lower among students who initiated smoking at earlier ages, which supports the explanation that older GHWs were no longer effective. Studies in Turkey that have examined the effectiveness of old (less harsh) warnings on packs find that they are usually ineffective [31, 32].

The results of this study should be interpreted in light of several limitations: Our sample was a convenience sample with participants recruited from four universities; hence, it may not be representative of university students in the country. The studied effects of package design elements may vary in the other cities of Turkey. Moreover, the cross-sectional nature of the data prevents us from interpreting the findings in a causal sense and observing behavioural changes over time. Another limitation is that, although participants could observe the images as long as they wanted, they viewed the packages on a screen rather than in hand; the effects of actual interaction may be different.

Another aspect of the PP regulation in Turkey was the choice of the black colour for the background, rather than the dark brown or dark green colour used in other countries. It is unknown whether and how the estimated effects in this study would change if the background were not black but another dark colour as in the other countries.

Despite these limitations, this study can motivate future quantitative and qualitative research to better understand the emotional impact of PP and GHW regulation and, equally importantly, any changes in the behaviour it generates. This can guide regulatory efforts to curb smoking during the critical years of tertiary education.

## Conclusions

This study used an online survey-based experiment among ever-smoker university students in Ankara, Turkey. No individual differential effect of PP or harsher GHWs was found on intentions to quit when respondents were exposed to images on a screen. However, PP and harsher GHWs were found to be effective in generating negative affect and avoidant responses.

Smoking incurs substantial health costs. The findings of this study imply that further work is needed for effective tobacco control among youth during critical years of tertiary education.

## Supplementary Information

**Additional file 1.**

## Data Availability

The datasets and variables generated and analyzed are available from the corresponding author upon reasonable request.
