# Supplementary material for: Perceptions of plain packaging and health warnings among university students in Turkey: a survey-based experiment
Source: BMC Public Health. 2023 Apr 28;23:779. doi: 10.1186/s12889-023-15637-4 (PMC10141903; doi:10.1186/s12889-023-15637-4)
Supplement: Supplementary file 1 — Additional file 1. [file 12889_2023_15637_MOESM1_ESM.docx]

**Additional file**

**Additional file 1 Table S1:** Means and 95% confidence intervals of dependent variables

|  | **Condition 1** | | **Condition 2** | | **Condition 3** | | **Condition 4** | | **Condition 5** | |
| --- | --- | --- | --- | --- | --- | --- | --- | --- | --- | --- |
| **n =** | 117 | | 120 | | 134 | | 131 | | 121 | |
| **Negative Affect** |  | |  | |  | |  | |  | |
| Afraid | 1.6[1.4-1.8] | | 1.8[1.6-1.9] | | 2.0[1.8-2.2] | | 2.3[2.1-2.5] | | 2.3[2.0-2.5] | |
| Angry | 1.5[1.3-1.7] | | 1.7[1.5-1.9] | | 1.8[1.6-2.1] | | 2.1[1.8-2.3] | | 2.1[1.8-2.3] | |
| Annoyed | 2.2[2.0-2.5] | | 2. 4[2.2-2.7] | | 3.0[2.7-3.2] | | 3.1[2.9-3.4] | | 3.2[3.0-3.5] | |
| Sad | 1.8[1.6-2.0] | | 2.1[1.9-2.3] | | 2.3[2.1-2.5] | | 2.6[2.3-2.8] | | 2.4[2.1-2.7] | |
| Disturbed | 1.9[1.7-2.2] | | 2.1[1.9-2.3] | | 2.6[2.3-2.8] | | 2.9[2.7-3.2] | | 2.9[2.6-3.1] | |
| Grossed-out | 1.5[1.3-1.6] | | 1.8[1.6-2.0] | | 2.4[2.2-2.7] | | 2.4[2.1-2.6] | | 2.7[2.4-3.0] | |
| Scared | 1.4[1.2-1.5] | | 1.7[1.5-1.9] | | 2.0[1.7-2.2] | | 2.1[1.9-2.4] | | 2.4[2.2-2.7] | |
| Guilty | 1.8[1.6-2.0] | | 1.8[1.6-2.0] | | 1.9[1.7-2.1] | | 2.1[1.9-2.4] | | 2.0[1.8-2.3] | |
| **Avoidant responses to health warnings** | |  | |  | |  | |  | |  |
| If I were smoking when I see one of these packs, I would stop smoking. | 1.2[1.1-1.3] | | 1.3[1.1-1.4] | | 1.4[1.3-1.6] | | 1.5[1.3-1.7] | | 1.4[1.3-1.6] | |
| I try to cover the pack in order not to see it. | 2.2[1.9-2.4] | | 2.2[1.9-2.4] | | 2.8[2.6-3.1] | | 2.9[2.6-3.1] | | 3.0[2.7-3.3] | |
| I try to hide it somewhere I cannot see. | 1.8[1.6-2.1] | | 2.1[1.8-2.3] | | 2.4[2.1-2.7] | | 2.5[2.2-2.8] | | 2.5[2.3-2.8] | |
| I would use a pack cover or a cigarette container in order not to see the pack. | 1.8[1.6-2.1] | | 2.0[1.8-2.2] | | 2.3[2.1-2.6] | | 2.6[2.3-2.9] | | 2.6[2.4-2.9] | |
| I prefer to buy a pack with another look. | 1.9[1.7-2.2] | | 2.3[2.0-2.6] | | 2.7[2.5-3.0] | | 2.9[2.6-3.2] | | 3.1[2.8-3.4] | |
| **Intentions to quit** |  | |  | |  | |  | |  | |
| Intention to quit within 6 months or sooner (%) | 4.3  [0.6-8.0] | | 9.2  [3.9-14.4] | | 9.0  [4.1-13.9] | | 12.2  [6.5-17.9] | | 13.2  [7.1-19.3] | |

**Note:** Students were randomly assigned to one of the five conditions. See the last column of Table 1 for the p-values of the tests of equality of explanatory variables across conditions.

**The Regulatory Environment in Turkey about Health Warnings on Cigarette Packages**

The Law No. 4207 enacted in 1996 (8) required a text warning about the detrimental health effects of smoking (“Disclaimer: Harmful to Health”) displayed on cigarette packs. The regulation in 2005 in the Official Gazette No. 25692 (9), required, in addition to keeping the earlier disclaimer, the general warnings “Smoking/Tobacco Kills” and “Smoking/Tobacco causes serious harm to you and those around you” and one of the following 14 text warnings to be displayed on cigarette packs:

*Smokers die younger; Smoking clogs the arteries and causes heart attacks and strokes; Smoking causes fatal lung cancer; Smoking when pregnant harms your baby; Protect children: don't make them breathe your smoke; Your doctor or your pharmacist can help you stop smoking; Smoking is highly addictive, don't start; Stopping smoking reduces the risk of fatal heart and lung diseases; Smoking can cause a slow and painful death; Get help to stop smoking: (telephone/postal address/internet address/consult your doctor/pharmacist); Smoking may reduce the blood flow and causes impotence; Smoking causes ageing of the skin; Smoking can damage the sperm and decreases fertility; Cigarette smoke contains benzene, nitrosamines, formaldehyde and hydrogen cyanide.*

Until 2010, cigarette packs in Turkey had only text warnings on them. Brand logos were visible and packs were printed in brand-specific colors. With the regulation in 2010 in the Official Gazette No. 27506 (10), combined health warnings were introduced by adding GHWs to the 14 text warnings that were already mandated to be displayed on cigarette packs. The pictorial warnings printed on the packages were selected from the pictures that were in the archive developed by the European Union.

Law No. 7151 in November 2018 (11) and the subsequent regulation in March 2019 (12) introduced PP and new stronger text and graphic health warnings. The new text warnings were the following:

*Smoking reduces blood flow and leads to impotence; Smokers die younger; Smoking causes throat cancer; Just one stroke can leave you helpless. Tobacco use is the biggest cause of stroke; Smoking causes fatal lung cancer; Protect children: don't make them breathe your smoke; Chronic bronchitis. Smoking causes chronic bronchitis, which makes it difficult to breathe every time you breathe; Smoking harms babies. Babies of mothers who smoked during pregnancy cannot grow well in the womb, and these babies are at increased risk of illness, disability, and death after birth; Smoking causes gangrene of the foot; Cigarette smoke contains carcinogenic substances such as benzene, nitrosamines, formaldehyde and hydrogen cyanide; Cigarette smoke harms babies. Smoking causes preterm birth in pregnancy, increasing the risk of death, illness and disability in these babies; Cigarette smoke kills children; Smoking causes fatal emphysema; Children see, children do. If you smoke, your child is twice as likely to smoke. Half of all premature deaths in lifelong smokers are due to smoking.*

**Additional Results: Sensitivity Analysis**

The sample that was used to estimate the regressions included all ever-smoker respondents, some of whom stated that they did not smoke in the last 30 days. Given that the outcome variables are more relevant to smokers, the regressions were estimated again in a restricted sample, in which those who did not smoke in the last 30 days were excluded. The new results are presented in Appendix tables A2 and A3.

The new results were very similar to the original ones, although they have less precision because of the reduction in the sample size. Combined warnings were associated with greater negative affect relative to text-only warnings (on packages with brand logos). New, stronger warnings were associated with greater avoidant responses relative to old warnings (on packages with brand logos). PP was associated with greater negative affect relative to branded packages (when old warnings were displayed). One new result was the estimation of greater avoidant responses to new warnings (relative to old warnings) on plain packages. So, in this sample, it was found that stronger warnings generated greater avoidant responses than old warnings when they were either on branded packages or on plain packages, unlike the earlier results which indicated that stronger warnings generated greater avoidant responses only when they were on branded packages. As in the earlier results, the new results did not indicate any significant association between individual package design elements and intentions to quit.

**Additional file 1 Table S2:** Regression models for the three outcome variables, comparing responses across experimental conditions (sample excludes those who did not smoke in the last 30 days)

|  | **(1)** | **(2)** | **(3)** | **(4)** |
| --- | --- | --- | --- | --- |
| **Outcome Variable: Negative Affect** | | | | |
| **Condition 1** | 0 | -0.21* | -0.41*** | -0.55*** |
| **Condition 2** | **0.21*** | 0 | -0.20 | -0.35** |
| **Condition 3** | 0.41*** | **0.20** | 0 | -0.14 |
| **Condition 4** | 0.55*** | **0.35**** | 0.14 | 0 |
| **Condition 5** | 0.66*** | 0.45*** | **0.25** | **0.11** |
| **Outcome Variable: Avoidant Responses** | | | | |
| **Condition 1** | 0 | -0.18 | -0.43*** | -0.40*** |
| **Condition 2** | **0.18** | 0 | -0.26* | -0.22 |
| **Condition 3** | 0.43*** | **0.26*** | 0 | 0.033 |
| **Condition 4** | 0.40*** | **0.22** | -0.033 | 0 |
| **Condition 5** | 0.68*** | 0.51*** | **0.25** | **0.28*** |
| **Outcome Variable: Intentions to Quit** | | | | |
| **Condition 1** | 0 | -0.043 | -0.057* | -0.025 |
| **Condition 2** | **0.043** | 0 | -0.013 | 0.019 |
| **Condition 3** | 0.057* | **0.013** | 0 | 0.032 |
| **Condition 4** | 0.025 | **-0.019** | -0.032 | 0 |
| **Condition 5** | 0.040 | -0.0033 | **-0.017** | **0.015** |
| **N** | 464 | 464 | 464 | 464 |

**Note:** There were 91, 93, 109, 90, and 81 respondents in the five experimental conditions, respectively. Columns in the table show estimates from different regressions. Estimates that were used to compare outcome variables across conditions are typed in bold. Coefficient estimates for reference categories are zero. Four versions of Model 2 were estimated, using in each version a different condition as the reference category. Condition 1: Brand logos + text, Condition 2: Brand logos + old text + old pictures, Category 3: Brand logos + new text + new pictures, Condition 4: PP + old text + old pictures, Condition 5: PP + new text + new pictures. *** p<0.01, ** p<0.05, * p<0.10

**Additional file 1 Table S3:** Effects of package design elements on outcome variables estimated through regression analyses by comparing across experimental conditions (Based on the results in table A2)

| Condition 1 vs. Condition 2 (Column (1)): | Combined health warning compared to text-only warnings  (on packages with brand logos) | **More** negative affect (at 10% statistical significance) |
| --- | --- | --- |
| Condition 2 vs. Condition 3 (Column (2)): | New warnings compared to old warnings  (on packages with brand logos) | **More** avoidant responses (at 10% statistical significance) |
| Condition 2 vs. Condition 4 (Column (2)): | PP compared to branded packages  (when old warnings are displayed) | **More** negative affect. |
| Condition 3 vs. Condition 5 (Column (3)): | PP compared to branded packages  (when new warnings are displayed) | No effect on any of the outcome variables |
| Condition 4 vs. Condition 5 (Column (4)): | New warnings compared to old warnings  (on plain packages) | **More** avoidant responses (at 10% statistical significance) |
